# Notch Signaling Suppression by Golden Phytochemicals: Potential for Cancer Therapy

**DOI:** 10.34172/apb.2024.035

**Published:** 2024-03-10

**Authors:** Masoumeh Kaveh Zenjanab, Nastaran Hashemzadeh, Sajjad Alimohammadvand, Masoumeh Sharifi-Azad, Elaheh Dalir Abdolahinia, Rana Jahanban-Esfahlan

**Affiliations:** ^1^Drug Applied Research Center, Tabriz University of Medical Sciences, Tabriz, Iran.; ^2^Pharmaceutical Analysis Research Center and Faculty of Pharmacy, Tabriz University of Medical Sciences, Tabriz, Iran.; ^3^Department of Medical Biotechnology, Faculty of Advanced Medical Sciences, Tabriz University of Medical Sciences, Tabriz, Iran.; ^4^Department of Oral Science and Translation Research, College of Dental Medicine, Nova Southeastern University, Fort Lauderdale, FL 33314, US.

**Keywords:** Cancer, Cell signaling, Notch signaling pathway, Natural compounds, Phytochemicals

## Abstract

Cancer is one of the main causes of mortality worldwide. Cancer cells are characterized by unregulated cellular processes, including proliferation, progression, and angiogenesis. The occurrence of these processes is due to the dysregulation of various signaling pathways such as NF-κB (nuclear factor-κB), Wnt/beta-catenin, Notch signaling and MAPK (mitogen-activated protein kinases). Notch signaling pathways cause the progression of various types of malignant tumors. Among the phytochemicals for cancer therapy, several have attracted great interest, including curcumin, genistein, quercetin, silibinin, resveratrol, cucurbitacin and glycyrrhizin. Given the great cellular and molecular heterogeneity within tumors and the high toxicity and side effects of synthetic chemotherapeutics, natural products with pleiotropic effects that simultaneously target numerous signaling pathways appear to be ideal substitutes for cancer therapy. With this in mind, we take a look at the current status, impact and potential of known compounds as golden phytochemicals on key signaling pathways in tumors, focusing on the Notch pathway. This review may be useful for discovering new molecular targets for safe and efficient cancer therapy with natural chemotherapeutics.

## Introduction

 Cancer is one of the most important diseases in industrialized and developing countries. In 2018, cancer contributed to 9.6 million deaths worldwide.^1^ Various environmental and genetic factors trigger mutations in susceptible cells, leading to growth, progression and ultimately cancer.^2^ Normal cells have mechanisms to suppress tumors. However, problems arise when these mechanisms and their functions are limited by a mutation in a gene that suppresses cancer.^3^ The body’s homeostasis is necessary for survival, and cell death is essential to control cell turnover. However, uncontrolled growth and tumors occur when cells cannot maintain the balance between survival and death.^4^ Signaling pathways play a critical role in maintaining the balance between proliferation, survival, and apoptosis of cells, and dysregulation of these pathways leads to uncontrolled growth of cells and metastasis to other parts of the body.^5,6^ Due to the high incidence of cancer, its treatment has been relatively unsuccessful. Current cancer treatment options include surgical removal and radiotherapy, usually followed by systemic chemotherapy.^7,8^ Available chemotherapeutic agents include anti-tubulin agents (taxanes), DNA-interacting agents (e.g., doxorubicin, cisplatin), antimetabolites (e.g., methotrexate), molecular targeting agents and hormones.^9,10^ While chemotherapy is one of the major cancer treatments, it has many disadvantages such as cancer recurrence, toxicity to nontargeted tissues, and drug resistance. These drawbacks can limit the use of chemotherapeutic agents. The search for new promising anticancer drugs with fewer side effects and better efficacy is essential to overcome the problems of current treatments.^8,11-13^

 Plant derivatives and phytochemicals are promising options for improving the efficacy of cancer patient treatment and eliciting minimum adverse effects.^14,15^ Some of these phytochemicals have significant anticancer effects.^16^ Many of the phytochemical components are part of the human diet. Thus, a long history of these phytochemical components including human exposure, good tolerance, low toxicity, and documented biological activities, is currently being evaluated to eliminate cancer.^17-22^

 Dysregulation of numerous signaling pathways, such as Notch, contributes to cancer progression and recurrence. The Notch signaling pathway plays an important role in stem cell survival, proliferation, renewal, cell differentiation, and cell fate determination during morphogenesis and development.^23^ Studies have shown that disruption of Notch pathway regulation contributes to carcinogenesis, angiogenesis, cancer stem cell (CSC) renewal, and resistance to chemotherapy. High levels of Notch ligands and receptors correlate with cancer progression and poor survival.^24^ In addition, the transcriptional activity of important target genes is regulated by the Notch signaling pathway via interactions with numerous other signaling pathways. The Notch signaling pathway has been shown to be a suitable therapeutic target for the treatment of various types of cancer. Researchers have demonstrated the anti-tumor properties of Notch inhibitors in various types of cancer.^25^

 The current review has classified a variety of phytochemicals and focuses on their effects on the different signaling pathways, with special attention to the Notch pathway. Also, the connection between the different signaling pathways, in particular the Notch pathway, with other signaling pathways is explained. In addition, the potential of these natural compounds for cancer therapy is discussed, which can be useful for the discovery of new molecular targets for safe and efficient cancer therapy with natural chemotherapeutics.

## Notch signaling pathway

 The Notch signaling pathway plays a critical role in cell fate, and mutations in this pathway lead to malignancy and drug resistance.^26^ The Notch signaling structure consists of the Notch receptors in humans (Notch 1-4), a transmembrane protein, and their ligands (Jagged1, 2 and delta-like ligands 1, 3 and 4).^27^ The adjacent cell ligands bind to the Notch receptors and γ-Secretase cleavages the receptor of Notch, then Notch intracellular domain (NICD) release. NICD translocates towards the nucleus, binds to numerous transcriptional regulators and induces apoptosis, and induces cell proliferation.^28-30^ In addition, Notch signaling during embryonic development plays an important role in lineage decision and stem cell maintenance. Depending on the organ and tissue, Notch signaling may play a different role, such as hematopoiesis in the fetus and in adulthood, or initiate terminal differentiation.^28^

**Figure 1:**
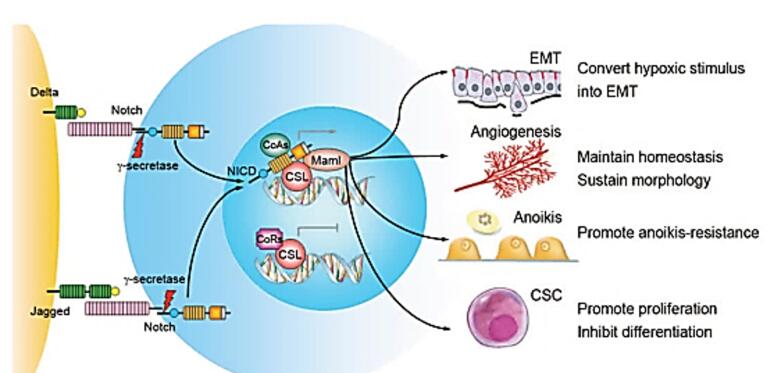
The pathway of Notch signaling and its roles in metastasis of cancers. Delta-like and Jagged families of ligands activate Notch receptors. NICD proteins, after proteolysis by γ-secretase, are transferred to the nucleus, bind to the DNA binding protein CSL, and take the place of corepressors (CoRs). NICD forms a complex with the coactivators (CoAs) and DNA binding protein CSL, which activate the transcription of Notch target genes. Notch signaling activation in the microenvironment of a tumor can increase tumor cell resistance to anoikis, epithelial-mesenchymal transition (EMT), maintenance of angiogenic homeostasis, vascular morphology, and self-renewal of cancer stem cells (CSCs). Adopted from Hu et al^31^ with permission from Springer

 Notch genes encode highly conserved transmembrane receptors that are involved in cell fate decisions (Figure 1).^31-33^ Signaling through this pathway depends on direct contact between neighboring cells expressing Notch receptors and ligands. Finally, downstream signaling leads to differentiation, proliferation, survival and regulation of cell fate specification.^33,34^

 Recent studies have shown that mutations in protein-coding genes lead to abnormal protein expression.^35^ Notch is one of the complex networks that play a key role in cell survival or death and also promote cancer cell growth and malignancy. The effects of this pathway on the tumor microenvironment (TME), such as matrix remodeling, are also known.^36^ Other signaling pathways, such as WNT, contribute to the promotion of Notch signaling pathways that can be targeted simultaneously.^37^ Several phytochemical and non-phytochemical compounds targeting the Notch signaling pathway have been reported. Some of these compounds are currently in clinical trials for a wide range of diseases, including γ-secretase inhibitors (RO4929097, MRK-003, MK-0752, PF-03084014, etc), immunotherapy (OMP-59R5, OMP-21M18, NRR1, NRR2, NRR3, A5226A, DLL1-Fc & JAG1-Fc) and MAM peptide antagonists (SAHM1).^38-40^ Milk phospholipids, Lactobacillus acidophilus, L. rhamnosus GG, γ-secretase inhibitors (dibenzoazepine, DAPT), 6-formylindolo(3,2-b)carbazole are examples of drug regulation of Notch whose effects on inflammatory bowel disease and colon cancer have been studied.^41^ Important phytochemicals in cancer therapy via Notch signaling suppression is presented in Table 1.

**Table 1:** Golden phytochemicals with potential to inhibit notch signaling for cancer therapy

**Phytochemicals**	**Chemical structure**	**Plant origin **	**group of natural substances**	**Biological effect**	**Ref**
Curcumin	**Figure:** 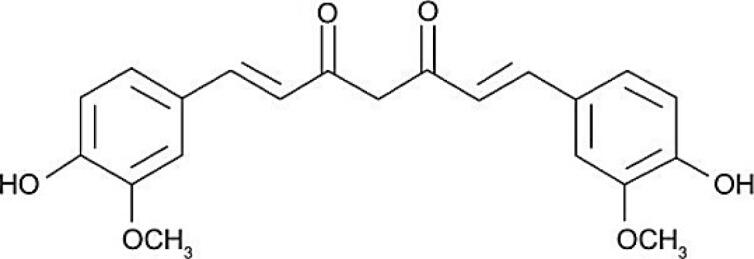	*Curcuma longa*	Diarylheptanoid	Treatment of several chronic diseases such as liver disease, inflammation, arthritis, metabolic syndrome, liver disease, obesity, neurological diseases, and also several cancers due to its anti-inflammatory and antioxidant actions	^42^
Genistein	**Figure:** 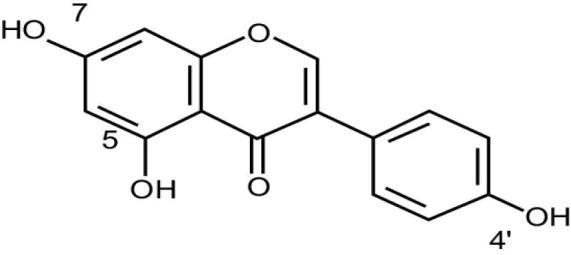	*Genista tinctoria*	Isoflavonoids	anti-tumor activity , impairment of angiogenesis in cancer cells, improvement of glucose metabolism, decrease of peri-menopausal and postmenopausal hot flashes, and modulation of antioxidant actions.	^43^
Glycyrrhizin	**Figure:** 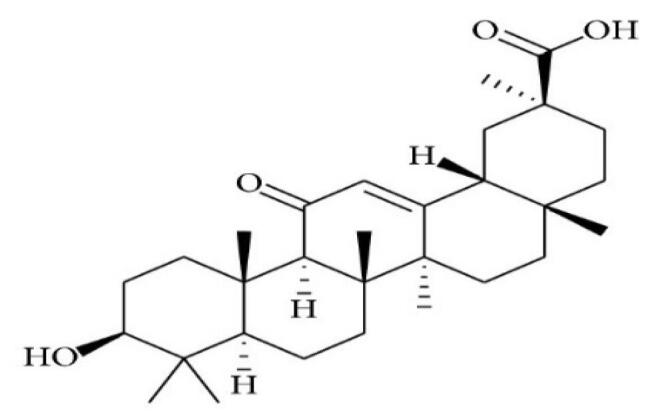	*Glycyrrhiza glabra*	Triterpene glycoside (saponin)	Anti-inflammatory, anti-cancer, anti-viral, and anti-oxidant activity	^44^
Cucurbitacin	**Figure:** 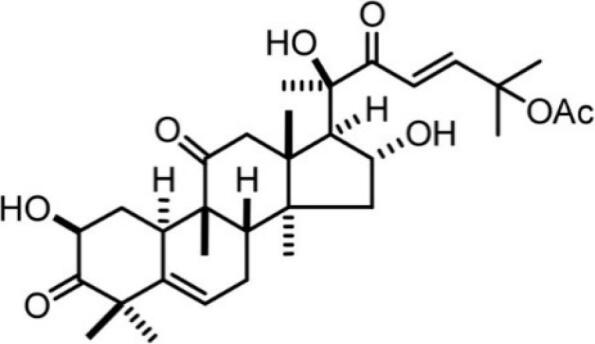	*Cucurbitaceae*	Triterpene	Anti-inflammatory and anti-cancer effects, treatment of cardiovascular diseases, and diabetes	^45^
Resveratrol	**Figure:** 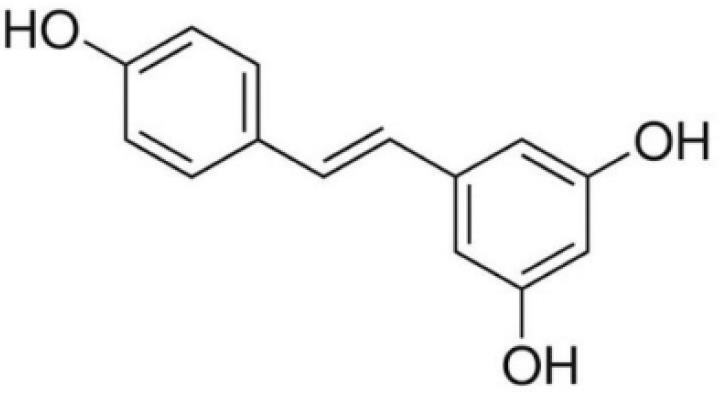	*Veratrum grandiflorum*	Stilbene	Antitumor, antioxidant, anti-viral, and phytoestrogenic effects	^46^
Silibinin	**Figure:** 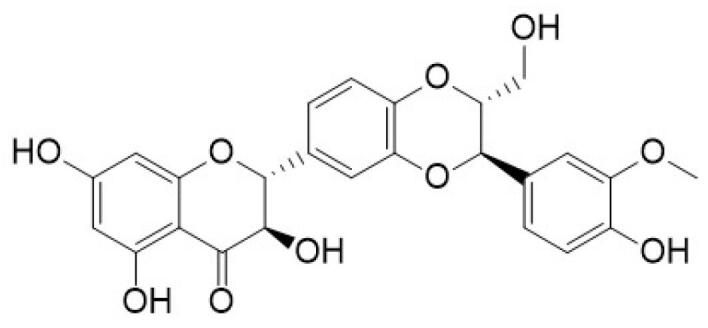	*Silybum marianum*	Flavonolignans	Anticancer, hepatoprotective, anti-inflammatory, and anti-fibrotic effects	^47^
Quercetin	**Figure:** 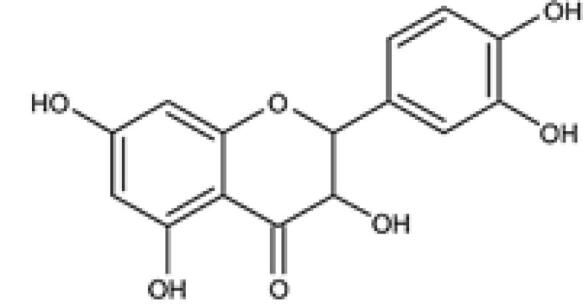	*Quercetum* (oak forest)	Flavonoid	Anti-oxidant, anti-cancer, anticarcinogenic, and antimicrobial activities	^48^
**Honokiol**	**Figure:** 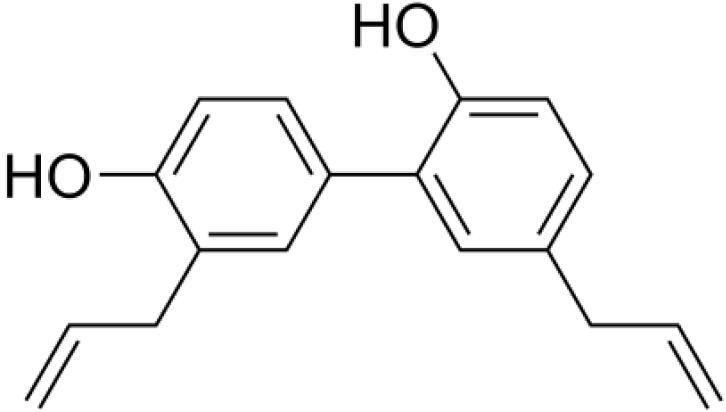	*Magnolia*	Lignan	Antitumorigenic, Antithrombotic, Anti-inflammatory, Anti-oxidant Anti-viral	^41^

## Phytochemicals’s effects against various cancers

 Since chemotherapeutic drugs are associated with significant side effects and toxicity, natural compounds such as phytochemicals show therapeutic benefits in various diseases such as cancer.^14,49^ The phytochemicals can be extracted from fruits and vegetables, and their antitumor activities influence the metabolism, proliferation and epigenetic modification of cancer cells.^16^

 About 50 % of the anticancer drugs approved in 2014 are derived from or directly extracted from natural products.^50^ Some important anticancer phytochemicals have been tested in vitro and in vivo for their anticancer activity. Phytochemicals have overlapping and complementary mechanisms to slow carcinogenesis by suppressing cancer cell survival and proliferation,^51^ reducing tumor invasion and angiogenesis,^52^ and scavenging free radicals.^53^ Phytochemicals exert a complex and wide-ranging influence on signal transduction pathways and various molecular targets such as suppressor proteins or downstream tumor activators,^54^ microRNAs,^55^ membrane receptors,^56^ transcription factors,^57^ kinases,^58^ cyclins and caspases.^51^

 Exciting research is related to endoplasmic reticulum (ER) stress, and the effect of natural compounds on apoptosis through ER stress is considered a suitable anti-cancer strategy.^59^ In addition, curcumin, and gallic acid have been used in breast, colon and ovarian cancer.^60^ The properties of important phytochemicals in cancer therapy that suppress Notch signaling are presented.

###  Curcumin

 Curcumin has been extracted from the rhizomes of *Curcuma longa*, the turmeric, since 1815 and has been taken into account by many scientists ever since. Due to its antioxidant and anti-inflammatory effects, curcumin may be beneficial in the treatment of various chronic diseases such as inflammation, arthritis, liver disease, metabolic syndrome, liver disease, obesity, neurological diseases and various cancers.^61,62^ Curcumin influences cell proliferation, growth, survival, apoptosis, migration, invasion and angiogenesis.^63,64^

 Another anti-cancer effect of curcumin is its influence on cyclin D1 levels, an important regulator of cell cycle progression. High levels of cyclin D1 are associated with the development and progression of cancer. Curcumin suppresses cyclin D1 by inhibiting nuclear factor-κB (NF-κB).^56^ NF-κB, a proinflammatory transcription factor, promotes the proliferation of breast cancer cells and controls the regulation of more than 500 different genes and protein expressions involved in the signaling pathway.^65,66^ In squamous cell carcinoma of the head and neck, curcumin has been reported to inhibit cell growth and stimulate apoptosis by suppressing NF-κB activity and expressing NF-κB-regulatory genes such as Bcl-2, cyclin D1, Cox-2 and MMP-9.^67^ Curcumin inhibits oral squamous cell carcinoma cell invasion by suppressing NF-κB activation, and studies suggest that NF-κB is regulated by the Notch signaling pathway in oral cancer.^68^ Notch signaling pathways are essential for regulating the balance between cell growth, differentiation, and apoptosis.^69^ Notch signaling pathways are involved in the development of cancers such as oral, pancreatic, prostate, breast, and many other cancers.^70-72^ Reducing Notch activity can be considered a promising approach to combating cancer in vitro and in vivo.^73^ Notch1 activity is essential for maintaining of NF-κB activity. Notch1 signaling pathways induce promoter activity and expression of multiple NF-κB subunits, as shown in mice in which decreased NF-κB activity leads to downregulation of Notch signaling pathways.^74^ Wang et al. investigated the effect of curcumin on the Notch1 signaling pathway in pancreatic cancer (BxPC-3 and PANC-1 cells). The results showed that curcumin reduced the transcription and translation of Notch1 as well as the expression of Hes-1, cyclin D1 and Bcl-X genes, which showed a decreasing trend compared to the control groups. In addition, this study showed that curcumin not only stopped cell growth but also induced apoptosis of BxPC-3 and PANC-1 cells. In this study, BxPC-3 cells were transfected with small-interfering RNA (siRNA) as a positive control, which inhibited the expression of the Notch1 signaling pathway. The NF-κB signaling pathway was measured by electrophoretic mobility shift assay (EMSA), which showed that Notch1 siRNA inhibited the DNA-binding activity of NF-κB and enhanced the effect of curcumin on NF-κB inhibition. This result proves the interaction between Notch1 and the NF-κB signaling pathway.^72^ Studies in oral cancer cells have shown that curcumin reduces Notch1 activity, leading to downregulation of NF-κB and its target genes such as Bcl-2, cyclin D1, VEGF, and MMP-9.^71^ The expression of HES1 proteins, which are the target genes of Notch1 and influence cell fate, was also decreased.^75^ Since curcumin has photodynamic properties, a combination of photodynamic therapy (PDT) and Notch receptor blockers (DAPT) was used. The results showed decreased proliferation, induced apoptosis, and blocked Notch signaling pathway by downregulating NF-κB and Notch1 expression.^76^

 The translation of messenger RNA (mRNA) is regulated by the eIF4F complex, which consists of the eukaryotic translation initiation factors eIF4A, eIF4E and eIF4G. A study has shown that the use of small interfering RNAs to knock down eIF4E in head and neck carcinomas leads to a decrease in cyclin D1 protein levels and inhibition of cell growth.^77^ When studying immortalized normal cells, Chakravarti et al. showed that all eIF4E proteins were downregulated after treatment with curcumin, which correlated with decreased cyclin D1 protein expression and inhibition of cell growth. They also found that immortalized normal and cancer cells had high levels of components of the eIF4F complex as well as proteins that activate eIF4F (Mnk1) or low levels of eIF4F inhibitory proteins (4E-BP1), as curcumin inhibits the growth of oral cancer cells.^78^

**Figure 2:**
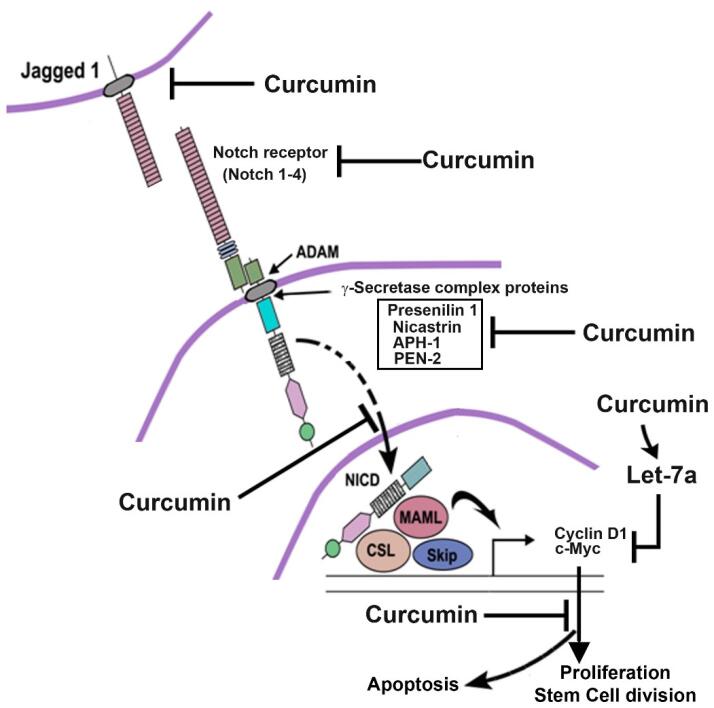
Influence of curcumin on Notch signaling in esophageal cancer. Curcumin inhibits Notch-1 and Jagged-1 receptor expression. Curcumin also prevents proteins of the γ-secretase complex and thus prevents the cleavage of the Notch receptor. Consequently, the NICD is not released and therefore does not translocate to the nucleus where it activates cyclin D1 and c-myc as the downstream target genes. This leads to the inhibition of cell proliferation and regeneration of stem cells while inducing apoptosis. Reprinted from Subramaniam et al^79^ under the terms of the Creative Commons Attribution License 4.0

 One study investigated the effect of curcumin on esophageal cancer cells. Curcumin reduced the size and number of esophageal spheres. In addition, treatment with curcumin resulted in reduced activation of Notch-1, the expression of Jagged-1 and its downstream target HES1. This reduced activation of Notch-1 was found to be due to the downregulation of key components of γ-secretase complex proteins such as nicastrin and presenilin 1. A combination of curcumin and a known γ-secretase inhibitor DAPT induced apoptosis and further reduced proliferation in esophageal cancer cells (Figure 2). Finally, curcumin decreased the expression of the microRNAs Notch1 miR-34a and miR-21 and upregulated the tumor suppressor miRNA let-7a.^79^

###  Genistein 

 Genistein is a phytoestrogen that belongs to the isoflavone family and is found in soybeans, chickpeas and other soy-based foods that are also used in herbal medicine.^80-82^ The daily intake of isoflavones is higher in people in Asian countries than in the Western world, and studies have shown that some specific cancers such as breast and prostate cancer are less common in Asian countries than in the Western world.^83^ A wide range of anti-cancer properties of genistein have been reported in numerous experiments, including interference with cell cycle control and apoptosis.^84^ However, genistein has many other beneficial effects on a variety of diseases. Due to its anti-inflammatory effects, it can cure diseases such as allergies.^85^ Receptor tyrosine kinases (RTKs) are activated by various peptide growth factors such as IGF (insulin-like growth factor), EGF (epidermal growth factor) and NGF (nerve growth factor) etc. RTKs cause phosphorylation of downstream pathway proteins leading to complicated cytoplasmic and nuclear events, such as phosphorylation of other proteins and activation of enzymes involved in cell growth, survival and differentiation.^86,87^

 The property of genistein to modulate PTKs was demonstrated in 1987. In vitro studies have shown that genistein inhibits EGF receptor activity by interacting with tyrosine–specific protein kinase-like EGF receptor and histone H2B as a phosphate receptor.^88,89^ MiR-34a is a miRNA involved in the suppression of cell growth by inhibiting Notch1 expression in pancreatic cancer.^90^ Xia et al revealed that treatment of pancreatic cancer cells with genistein resulted in increased re-expression of miR-34a in cancer cells and down-regulation of Notch1. They also found that treatment of pancreatic cancer cells with miR-34a and genistein significantly reduced Notch1 levels compared to genistein alone.^91^ Zhou et al reported that genistein decreased the expression of Notch1 signaling protein and also induced the expression of Bax/Bcl-2, caspase-8 and caspase-3 in HT-29 cells (colon cancer), which decreased cancer cell growth and increased apoptosis.^92^ Some studies have shown that genistein suppresses the activity of Akt, leading to inactivation of the downstream signaling pathways, NF-κB. In addition, genistein directly inactivates NF-κB and subsequently reduces cancer cell growth.^93^

###  Quercetin

 Quercetin is a subclass of polyphenolic flavonoids that are abundant in natural products such as vegetables, fruits, and cereals, mostly in the form of glycosides.^94^ There is evidence that quercetin has numerous biochemical effects such as antioxidant, anticancer, anticarcinogenic, and antimicrobial activities.^95^ CSCs have unique properties such as self-renewal and differentiation potential that may be very similar to the properties of stem cells, such that many cancer cells arise from normal stem cell transformation.^96,97^ Extensive research has also shown that CSCs lead to cancer cell resistance to treatment, recurrence after treatment, and tumor regeneration.^98^ Li et al. documented that quercetin has a dramatic anticancer effect by suppressing the Notch signaling pathway in colon CSCs. These studies showed that protein levels of Jagged-1, all five proteins of the γ-secretase complex, and cleaved Notch1 decreased dramatically in colon cancer cells treated with quercetin plus radiotherapy.^99^ Experiments with human colon cancer xenografts in the BALB/c mouse model also showed that synergistic treatment of CSCs with quercetin and radiation was more efficient in reducing tumor volume than quercetin or radiation alone.^99^ The division of CSCs by self-renewal leads to two identical daughter cells (symmetric cell division) or two abnormal daughter cells (asymmetric cell division).^100^ Symmetric cell division leads to the growth and regeneration of tumor cells, whereas asymmetric cell division leads to the maintenance of the number of CSCs.^101,102^ The upregulation of Notch signaling is a trigger for symmetric division and eventually leads to cancer.^103^ Clifford et al reported that quercetin remarkably upregulates miR-200b-3p, a non-coding RNA that directly targets the 3’UTR of Notch1, and causes inhibition of Notch signaling by performing a dual-luciferase reporter assay in pancreatic ductal adenocarcinoma CSCs. In addition, they transfected miR-200b-3p into cancer cells, and the result of this experiment showed that treatment of CSCs with miR-200b-3p and quercetin caused the symmetric division of CSCs to change to asymmetric and inhibited not only the self-renewal but also the differentiation of CSCs by inhibiting Notch signaling.^104^

 Treatment of the AGS human gastric adenocarcinoma cell line with quercetin in combination with a low dose of SN-38 (inhibitor of DNA topoisomerase I) enhanced the anticancer effect of SN-38 by decreasing β-catenin protein levels and increasing apoptosis. In addition, treatment with a combination of SN-38 and quercetin in the AGS xenograft mouse model showed that the expression of cyclooxygenase-2 and markers of epithelial-mesenchymal transition, such as ITGβ6 and Twist1, was remarkably reduced compared to SN-38 treatment.^105^

###  Silibinin

 The natural flavonoid silibinin or silybin is the main bioactive compound of silymarin, which is isolated from the milk thistle plant (*Silybum marianum*), and the extraction of silibinin is used in traditional medicine to treat various diseases.^106,107^ The numerous beneficial properties of silibinin include hepatoprotective, antioxidant, anti-inflammatory, chemopreventive and many other properties.^108^ In addition, many studies have shown the anti-cancer effects of silibinin against various cancers such as lung, bladder, prostate, breast, lung, skin, colon and ovarian cancer.^109^ Kim et al reported that silibinin has a promising anticancer effect on breast cancer. According to their experiments, silibinin treatment induced apoptosis in MCF7 and MDA-MB-231 cells by increasing the formation of ROS and downregulating the expression of Notch1 mRNA in both cancer cells.^110^ Zhang et al investigated the effects of silibinin on human HepG2 cells (hepatocellular carcinoma). The result showed that silibinin treatment decreased the migration and adhesion ability of HepG2 cells, increased the generation of ROS, caspase3 activity and apoptosis. In addition, the combined treatment with silibinin and transfection of Notch1 siRNA onto HepG2 cells was found to decrease NICD levels and Notch signaling. In addition, Bax was upregulated and the survivin gene was downregulated compared to cells treated with silibinin or transfected with Notch1 siRNA.^111^ Chang et al treated line 786-O renal carcinoma with silibinin, and their results showed that urokinase plasminogen activator (u-PA), metalloproteinase (MMP) -2, -9 and MAPK pathway protein expression decreased and tumor weight was reduced.^112^ Studies on human ovarian cancer cells showed that silibinin inhibits ERK and Akt and reduces tumor growth.^113^ Non-small cell lung cancer was treated with silibinin. The result showed that silibinin disrupts cell proliferation by stimulating G0/G1 cell cycle arrest and apoptosis, and prevents tumor angiogenesis, invasion and migration. Silibinin regulated phosphorylated EGFR expression by binding to its receptor and then inhibited downstream targets and JAK2/STAT5 and PI3K/AKT signaling pathways, leading to a reduction in cancer rate.^114,115^

###  Resveratrol 

 Some spermatophytes, such as grapevine, produce resveratrol, a natural phytoalexin. Resveratrol is a stilbenoid found in various plants such as grapes, peanuts, and berries that protects them from environmental stress and pathogenic attack when damaged; red wine also contains high levels of resveratrol.^116-118^ Many studies have shown that resveratrol has important pharmacological properties, such as prevention of heart disease, slowing the aging process, alleviating diabetes, reducing inflammatory stress, lowering blood lipids, resistance to lipid peroxidation, usefulness in curing coughs and asthma, resistance to pathogenic microorganisms, and the unique ability to fight various types of cancer by suppressing the initiation, growth and progression of cancer and inducing apoptosis.^119-121^ In addition, resveratrol has antioxidant potential due to the three hydroxyl groups that interfere with intracellular redox signaling.^122^ Dong et al found that MDA-MB-231 cells (breast cancer cells) showed significantly decreased expression of Notch1 protein and decreased expression of Jagged1 ligand after treatment with different concentrations of resveratrol. In addition, the expression of the Hes5 protein was significantly decreased compared to the control group, and a downregulation of the Delta-like 4 ligand (Dll4) was also observed.^123^ Dll4/Notch signaling plays an important role in embryonic vascular development. Downregulation of DLL4 leads to decreased expression of EphrinB2 and HEY1 and prevents proliferation, migration, and network formation of endothelial cells, all of which play important roles in tumor angiogenesis.^124^ The study on human osteosarcoma cells treated with resveratrol showed it led to the inhibition of JAK2/STAT3 signaling reduced cell viability, self-renewal ability, and tumorigenesis. In addition, inhibition of JAK2/STAT3 signaling led to a decrease in the osteosarcoma stem cell marker CD133. The expression of Bcl-2 was increased, so resveratrol led to a decrease in the CSC subpopulation of osteosarcoma and inhibited the self-renewal ability of these cells.^125^ Li et al reported that resveratrol in combination with artemisinin has a stronger anti-cancer effect compared to treatment with artemisinin and resveratrol alone. In their experiment, cell apoptosis was examined and it was found that the number of apoptotic cells in HeLa cells increased after synergistic treatment with resveratrol and artemisinin compared to the treatment group alone. In addition, DCF fluorescence staining showed that HeLa cells treated with the combination of artemisinin and resveratrol increased ROS production and the migration rate decreased significantly in HeLa cells treated with artemisinin and resveratrol.^126^

###  Cucurbitacin 

 Cucurbitacin (cucurbitacin A-T) is one of the natural compounds isolated from some cucurbits such as bitter melons, zucchini, and pumpkins.^127^ Cucurbitacin acts on cancer cells by inducing apoptosis in cancer cells, especially in CSC, and arresting the cell cycle in G2/M, which inhibits proliferation.^128^ One of the unique properties of cucurbitacin is to affect the expression of CD44, one of the specific markers for cancer cells, and to increase apoptosis in head and neck cancer by decreasing STAT3 signaling, whereby STAT3 contributes to tumor cell progression, migration and survival.^129^ It has been suggested that cucurbitacin E suppresses MAPK kinases, a signaling pathway, and inhibits angiogenesis in cancer cells Cucurbitacin B was reported to inhibit signaling pathways such as Wnt/β-catenin and Hippo- YAP in lung cancer and colon cancer, respectively.^130^ It was shown that treatment with cucurbitacin B and I resulted in the downregulation of Notch signaling pathway including receptors, ligands, γ-secretase, and target genes. The decrease in Notch signaling expression led to a decrease in invasive behavior and poorer survival, which correlates with the decrease in epithelial-to-mesenchymal transition (EMT) in colon cancer cells.^131^ Dysregulation of Notch1,2 and their ligands is associated with colon cancer, and Notch3 and the ligands Jagged-1 and Dll-4 are also dysregulated in the aggressive phenotype of xenografts.^132^ Colon cancer cells exhibiting high Notch signaling activity were treated with Bcl-2 N-[N-3,5-difluorophenacetyl]-l-alanyl-S-phenylglycine methyl ester (DAPM) inhibitors of γ-secretase and showed a loss of subpopulations of cancer cells in xenografts.^133^ The interaction of cucurbitacin I and B with Notch1 has been reported to alter protein conformation and suppress the growth of colon cancer cells and stem cells.^128^

###  Glycyrrhizin 

 Glycyrrhizin is extracted from licorice (*Glycyrrhiza glabra* Linn), one of the most important medicinal plants known for its antioxidant, antiviral, antidiabetic and anti-inflammatory properties.^134^ In addition, the antitumor role of glycyrrhizin is confirmed by slowing down the cell cycle in G0/G1, increasing the production of intracellular ROS and inducing apoptosis in cervical cancer. Glycyrrhizin modulates Notch signaling through the action of Notch1 and the ligand Jagged-1, which reduces the expression of its downstream target gene HES-1 and cyclin D1, which is one of the important proteins that activate NICD in cervical cancer. This study also showed that down-regulation of Notch signaling by glycyrrhizin resulted in increased up-regulation of pro-apoptotic proteins such as Bad and Bax and down-regulated expression of anti-apoptotic proteins such as Bcl-2.^135^ Treatment of CaSki cervical cancer cells with glycyrrhizin has been shown to suppress proliferation, lead to mitochondrial dysfunction and decrease Notch1 mRNA expression.^136^ High mobility group box 1 (HMGB1), which triggers EMT, was targeted by glycyrrhizin in prostate cancer and glycyrrhizin was shown to inhibit HMGB1.^137^

## Conclusion

 Cancer is considered a critical health condition that is highly associated with death. Therefore, it is clear that the development of the best treatment strategies seems necessary. Among the various cancer treatment approaches, targeting a different part of the signaling pathways that play a key role in maintaining the balance between survival and death by somatic cells could be promising for cancer therapy. These cancer therapies targeting hormone receptor signaling, RTKs, the MAPK pathway, NF-κB, cyclin-dependent kinases, etc. are applicable and have received regulatory approval. As discussed in this review, the Notch signaling pathway has special properties for targeting different types of cancer and can be targeted with different strategies, such as cleavage inhibitors, γ-secretase inhibitors, γ-secretase modulators, antibodies against ligands and receptors, and transcription blockers. Natural products with unique properties are potential substitutes for chemotherapeutic agents and provide adequate therapeutic results. They can induce cell death mediated by signaling pathways, autophagy and apoptosis, reduce chemoresistance and inhibit the drug efflux pump. Although these natural agents are beneficial, there are still some concerns, such as the narrow therapeutic window and liver toxicity, which need to be further investigated.

 In the last century, cancer for which there is no definitive treatment has been the most common factor leading to human death. Therefore, there is an urgent need for therapeutically effective drugs that are less toxic to the body, such as natural products. This review provides a rationale and a potential target for the treatment of cancer by herbal anticancer agents.

## Acknowledgments

 RJE is supported by Drug Applied Research Center, Tabriz University of Medical Sciences, Tabriz, Iran, grant number 62674.

## Competing Interests

 The authors declare no conflict of interest.

## Ethical Approval

 Not applicable.
